# Clinical and mutational spectrum of patients with congenital lipoid adrenal hyperplasia in Southeast Asia

**DOI:** 10.1186/1687-9856-2015-S1-O55

**Published:** 2015-04-28

**Authors:** Kansuda Ariyawatkul, Patra Yeetong, Somchit Jaruratanasirikul, Kah Yin Loke, Pairunyar Nakavachara, Chawkaew Kongkanka, Taninee Sahakitrungruang

**Affiliations:** 1 Chulalongkorn University, Bangkok, Thailand; 2 Prince of Songkla University, Songkhla, Thailand; 3 KTP- National University Children's Medical Institute, National University Hospital, Singapore; 4 Siriraj Hospital, Mahidol University, Bangkok, Thailand; 5 Queen Sirikit National Institute of Child Health, Bangkok, Thailand

## Aims

Mutations in Steroidogenic Acute Regulatory protein (StAR) cause congenital lipoid adrenal hyperplasia (lipoid CAH), characterized by absent steroidogenesis, potentially lethal salt loss, 46,XY sex reversal and massively enlarged adrenals engorged with cholesterol esters. Nonclassic lipoid CAH is a recently recognized disorder caused by StAR mutations that retain partial function. We aim to delineate the clinical and mutational spectrum of StAR mutations in patients with lipoid CAH.

## Methods

The entire coding regions of the StAR gene were assessed by polymerase chain reaction and sequencing analysis.

## Results

There were 10 patients of lipoid CAH had mutations in the StAR gene with 5 novel mutations (p.P230L>WfsX, IVS6-1G>A, IVS3+(2-3)insT, p.W147R, p.Q264R). Eight patients had classic lipoid CAH presenting with adrenal crisis during early infancy (range of onset 3-11 months of age). Two siblings had nonclassic phenotypes with later onset adrenal insufficiency without disordered sex development. Adrenal enlargement by imaging was demonstrated in only 3 cases of classic lipoid CAH. The functional studies of novel StAR mutations are being under investigation.

## Conclusion

StAR mutations may not be rare in Southeast Asian population. There is a broad clinical spectrum of StAR mutations varying from early onset adrenal insufficiency to late onset of glucocorticoid deficiency with only mild defects in mineralocorticoid and sex steroid synthesis. Adrenal gland enlargement is not pathognomonic for lipoid CAH.

